# Dynamic scan paths investigations under manual and highly automated driving

**DOI:** 10.1038/s41598-021-83336-4

**Published:** 2021-02-12

**Authors:** Jordan Navarro, Otto Lappi, François Osiurak, Emma Hernout, Catherine Gabaude, Emanuelle Reynaud

**Affiliations:** 1 EMC (Laboratoire D’étude Des Mécanismes Cognitifs), University Lyon 2, Bron, France; 2 Institut Universitaire de France, Paris, France; 3 Traffic Research Unit, University of Helsinki, Helsinki, Finland; 4 IFSTTAR-TS2-LESCOT, University of Lyon, Bron, France

**Keywords:** Psychology, Human behaviour, Perception, Sensorimotor processing

## Abstract

Active visual scanning of the scene is a key task-element in all forms of human locomotion. In the field of driving, steering (lateral control) and speed adjustments (longitudinal control) models are largely based on drivers’ visual inputs. Despite knowledge gained on gaze behaviour behind the wheel, our understanding of the sequential aspects of the gaze strategies that actively sample that input remains restricted. Here, we apply scan path analysis to investigate sequences of visual scanning in manual and highly automated simulated driving. Five stereotypical visual sequences were identified under manual driving: forward polling (i.e. far road explorations), guidance, backwards polling (i.e. near road explorations), scenery and speed monitoring scan paths. Previously undocumented backwards polling scan paths were the most frequent. Under highly automated driving backwards polling scan paths relative frequency decreased, guidance scan paths relative frequency increased, and automation supervision specific scan paths appeared. The results shed new light on the gaze patterns engaged while driving. Methodological and empirical questions for future studies are discussed.

## Introduction

Complex visual scene exploration is a task we undertake almost all the time in our everyday life, and it is present in most tasks involving orientation and locomotion in complex surroundings, such as cooking, childcare or driving in traffic. The exploration of a visual scene requires gaze control, i.e. “*the process of directing fixation through a scene in real time in the service of ongoing perceptual, cognitive and behavioural activity*”^1^. Gaze control is under the influence of bottom-up stimulus features (i.e. optical information in the current visual input) and top-down knowledge (i.e. previously memorized information, goals), and a combination of both automatic and intentional processes. We can decide to gaze at a particular element in a visual scene for a longer time (e.g. our child) but most of the time our eyes are also scanning the environment, without the individual fixations reaching the level of consciousness. This is actually the default situation, as we are not aware that most of the time our eyes dart from one part of the scene to another about three times a second our whole waking life for review see^2^.

Locomotion is an activity involving tight visuo-motor coordination between gaze control and target achievement and avoidance. Driving is one everyday example of tool-mediated locomotion that critically relies on frequently sampling relevant visual cues (more so than for example walking). Gaze control while driving has been investigated for about 50 years. Many early studies emphasized the close coupling of gaze and hand movements engaged to steer the vehicle^3–6^. Specifically, movements of the eyes precede the movements of the hands by about a second while negotiating bends^7–9^.

However, such eye-hand coordination does not fully describe the complex visual strategies in car driving^10,11^. Driving a vehicle implies a variety of cognitive functions, engaging multiple levels of perceptual analysis and cognitive control^12,13^, relying on different neural areas see^14^ for a meta-analysis. Gaze, however, can only be directed at one object or location at a time, which implies flexible sequential control to allow different perceptual, cognitive and motor processes to share gaze time.

In the present study, we approached gaze strategies in driving from the point of view of the sequences of fixations (over an extended period of time). Considerable experimental knowledge has been gained on gaze behavior while driving^3,7,9,15–19^ and a number of models of how lateral or longitudinal control relates to visual behavior ^e.g.^^12,20–25^; but very little is still known of the *sequence* of visual explorations inside the driving scene, or the underlying mechanisms^26^.

One challenge is the dynamic nature of that situation. In lab tasks, the sequential organization of eye movements is traditionally studied by dividing a display into fixed Areas Of Interest (AOIs) and registering the transitions of gaze from one AOI to another. This reduces gaze position time series data into a discrete sequence of AOI glances or *scan paths*^27,28^. The properties of scan paths, including the overall similarity of two scan paths can be analyzed quantitatively using a variety of methods developed mainly in the reading and picture-viewing literature^29^.

However, extending the scan path analysis approach to dynamic tasks such as driving is not entirely straightforward. First, in a dynamic task it may not make sense to partition the visual field into fixed AOIs. One may of course treat the windscreen and mirrors, for example as fixed (in the vehicle frame of reference) AOIs, but because the visual content of the AOIs will change over time, it would often be desirable to further partition the windscreen into smaller AOIs based on the scene visible through the windscreen at any moment in time. These AOIs should, moreover be dynamic, as it is objects and locations of interest (e.g. a car in an intersection, a traffic sign) that are the natural semantic units of drivers’ visual exploration (rather than specific portions of the windscreen). In this case the different AOIs sizes, shapes and/or locations are changing at each time frame^30–32^. Second, natural behavior consists of extended action sequences, not trials of successively presented “stimulus events” with clear onset and offset that would give natural begin and end points for scan paths. It would probably not be very informative to compare, say, the similarity of two scan paths of an hour’s drive through a city, as the “stimulus sequences” are completely different. Rather, what one should be looking at are subsequences of some meaningful grain size. To the best of our knowledge there are no standard quantitative concepts and methods developed for such analyses. Here, we perform dynamic scan paths analysis to investigate sequences of visual exploration and shed light on visual control while driving in a car-following task.

A second objective is to investigate how driving automation is impacting the sequences of visual exploration. Driving automation is an important societal issue raising a variety of research questions^33,34^, as each automation level introduction creates difficult-to-predict changes in the complex human–machine cooperation system^35–37^. The currently available highest form of automation, referred as Highly Automated Driving (HAD), is able to manage both lateral and longitudinal control of the vehicle see^38^ for a review. However, in case of automation malfunction, or driving situations automation is not able to handle, take-over requests are required^39,40^. Here, dynamic scan paths analysis was carried out to refine the understanding of the impact of HAD on visual exploration.

## Methods

### Participants

Sixteen licenced car drivers (9 females) aged 19 to 26 years (M = 22 years; SD = 1.9 years) with driving experience ranging from 1.2 to 8.5 years (M = 4 years; SD = 1.7 years) participated in the experiment. All were French drivers used to drive on the right-hand side. All participants had normal uncorrected vision and no cognitive disorders. Written informed consent was obtained from all participants. The Ethics Committee of Department of Psychology of Lyon 2 Lumiere approved the study and the methods were carried out in accordance with the relevant guidelines and regulation.

### Material

**Figure 1:**
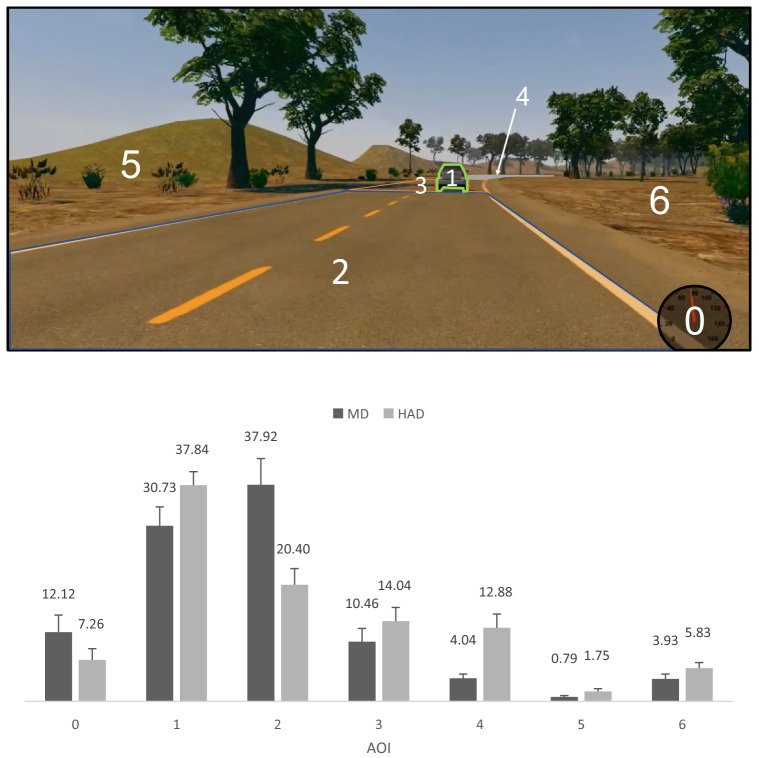
Top. Representation of the seven AOIs used for gaze position analyses. Note that the width of the background image was cropped (Photograph JN). Bottom. Relative frequency (in percentage) of AOI visits under Manual Driving (MD) and Highly Automated Driving (HAD). Error bars represent one standard error.

The experiment was conducted on a fixed-base three-screen Dell P2219H monitors, providing a horizontal field of view of about 145° (at a 3 * 1920 * 1080 resolution), with a refresh rate at 60 Hz. The driving simulator was developed by the University of Sherbrooke with C++ language and uses the OGRE 1-7-4 open-source 3D rendering engine with the Direct3D 9 API. The computer running this software was equipped with an Intel Xeon W-2133 processor running at 3.6 GHz, and a graphics card Radeon Pro WX-7100 with 8 Gb of GDDRS memory, clocked at 1.75 GHz see^41^ for additional information. The simulator included an adjustable seat (JCL Sim Racing), a steering wheel with force feedback, accelerator and brake pedals (logitech G27). A speedometer was displayed at the bottom right of the visual scene (Fig. 1).

Drivers’ gaze behaviours were recorded by means of an eye-tracker (iView X head-mounted, SensoMotoric Instruments) using a 9-point calibration procedure prior to the experiment with a sampling rate of 50 Hz. To ensure high quality recordings, (i) the helmet supporting the eye-tracker was adjusted and fastened to participants’ heads, (ii) visual scene and eye cameras were adjusted, (iii) participants were asked to successively gaze at the 9-point schema used to calibrate the eye-tracker, (iv) the quality of gaze tracking was visually checked by the experimenter over the 9-point schema through the video, continuously displayed by the eye-tracking system. Four persons were not invited to continue the experiment because the quality of the calibration was not sufficient. Calibration accuracy was assessed by a visual check to ensure that all calibration fixations made by the participants were inside a circle of a 2° radius centered on the calibration points. Although the accuracy of the calibration was not recorded for that experiment, a calibration quality check was performed on an additional sample of eight participants who were not part of the original study. Using the same calibration methodology, a calibration validation was recorded and revealed a mean accuracy of 0.9° ± 0.3°.

### Procedure

After a 10-min familiarisation drive including a car-following task, participants were required to follow a leading vehicle on a winding road. The road was composed of a succession of short straight sections and ten bends (5 left bends and 5 right bends with mean radii of curvature ranging from 44 to 313 m; M = 121 m; SD = 71.7 m). The road was a two-lane rural road without traffic signs or other traffic. Participants were instructed to follow the leading vehicle at a close but safe distance, as they would do in real life. The car-following task lasted for 5 min. The initial leading vehicle speed was set at 20 kph allowing the driven vehicle to catch up, then the leading vehicle speed varied according to a sine wave pattern oscillating between 50 and 90 kph with a period of 30 s. A fully detailed description and visualization of the car-following task can be found in Navarro et al.^41^. Driving behaviours analysis showed that the average time that separates the driven from the leading vehicle was of 1.51 s (SD = 0.57) and that the average standard deviation of that time was of 0.66 s (SD = 0.45). After the completion of the car-following task, participants had to keep driving manually for about 10-min in urban, peri-urban and highway environments. The Manual Driving (MD) condition then came to an end.

After a break of 3–5 min, the eye-tracker 9-points calibration was checked and participants were instructed that they will now drive the same simulated drive (i.e. car-following and urban, peri-urban and highway drive), but under Highly Automated Driving condition (HAD). Participants were informed that under HAD, automation will undertake both lateral and longitudinal control without any physical action on the steering wheel required. They were also instructed to keep monitoring the driving environment. In fact, they faced a replay of their own MD condition. As a consequence, the dynamic driving scene was exactly the same in MD and HAD.

Only the car-following task in the MD and HAD conditions was analysed. During that car-following section the leading vehicle was the only dynamic object present in the environment.

### Data analysis

The analysis of gaze behaviours in dynamic environments such as driving is challenging as the contents of the visual scene depend upon previous operator behaviour, and thus is not under experimenter control.

Gaze positions were classified into seven different dynamic AOIs, numbered from 0 to 6, in Fig. 1. AOI 0 corresponds to the speedometer; AOI 1 to the leading vehicle; AOI 2 to the section of the road located between the driven vehicle and the leading vehicle; AOI 3 to the road section around the leading vehicle, AOI 4 to the road section further away that the leading vehicle and AOIs 5 and 6 to the left and right of the road respectively. This classification was performed in order to distinguish between Near Fixations (NFs) at the road (AOI 2), Guiding Fixations (on [AOIs 1] and around [AOI 3] the leading vehicle; hereafter respectively referred as Leading Vehicle Fixations [LVFs] and Guiding Fixations [GFs]), Look Ahead Fixations (LAFs, AOI 4), fixations at the speedometer (AOI 0) and off the road (AOI 5 and 6).

Unless dynamic AOIs can be determined by the simulator software, eye-movement investigations require laborious image per image analyses. Here, image per image analyses were performed manually by an experimenter and checked by another experimenter. A minimum fixation duration of 80 ms was used.

Before considering scan paths, the relative frequency of AOI visits was computed (Fig. 1, bottom). The observed frequencies of the seven AOIs were significantly different from a uniform distribution under both Manual Driving (χ^2^ (6) = 41.17, *p* < 0.001) and Highly Automated Driving (χ^2^ (6) = 27.41, *p* < 0.001). Although representing a small surface on the visual scene, the leading vehicle (AOI 1) attracts about a third of the visits. In contrast, large visual scene surfaces (AOI 5 & 6) attracts very few visits. This indicates that it is the relevance of the information available in the AOI rather than its surface area that matters. Under HAD and compared to MD, fewer visits were payed to AOI 2 (χ^2^ (1) = 7.43, *p* < 0.05) and a non-significant trend toward more visits to AOI 4 (χ^2^ (1) = 5.04, *p* < 0.09) were recorded. No significant differences between MD and HAD were observed for the other AOIs (*p* > 0.05), in all cases the Benjamini–Hochberg procedure for correcting multiple comparisons was used^42^). In brief, HAD reduces the amount of NFs in favour of LAFs, in line with previous observations^19,43^. Although of interest, such an analysis does not provide any insight on the relationships between the different AOIs. The heart of this contribution is precisely to focus on the order of fixation of the different AOIs in order to identify meaningful fixation patterns.

Scan paths were then computed on the AOI data to investigate the sequences of visual exploration. A scan path is here defined as a succession of gaze fixations at different AOIs, regardless of the duration and the number of saccades and fixations inside a single AOI. The scan path length is defined as the total number of consecutive AOIs contained in a single scan path. Scan paths were computed using a window sliding over the whole sequence of gaze fixations.

Scan paths with lengths from 2 to 8 were initially considered. With the shortest scan path length (2 consecutive AOIs), 7 AOIs × 6 AOIs = 42 different AOI permutations were possible. With the longest scan path length (8 consecutive AOIs), 7 AOIs x (6^7) AOIs = 1 959 552 different AOI permutations were possible.

Scan paths analyses started at the initiation of the car-following task and stopped at the end of that same task. Inside that sequence, all possible scan path start positions were considered (i.e. first AOI, second AOI, third AOI, …, until the last AOI minus the scan path length considered).

Pearson’s chi-squared tests were used for statistical inferences.

### Scan path length selection

**Figure 2:**
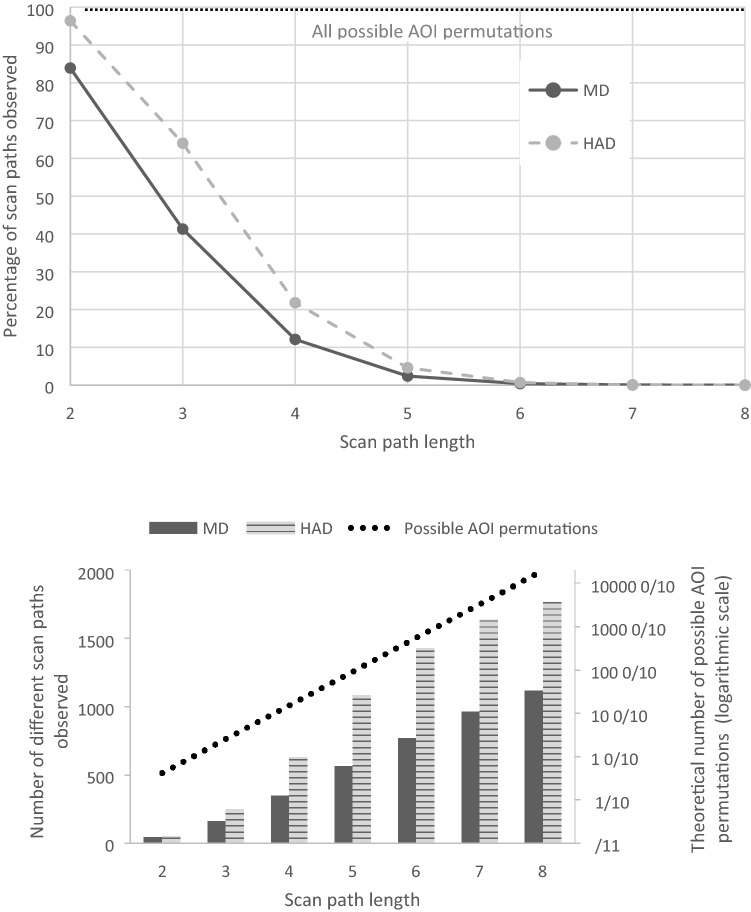
Top. Percentage of different scan paths observed relative to the theoretical number of possible AOI permutations (100%, dotted line on the figure) along with scan path length. Bottom. Number of different scan paths observed depending on scan path length and theoretical number of possible AOI permutations. Note that the scale on the left is linear, while the scale on the right is logarithmic. For Manual Driving (MD) in dark grey and Highly Automated Driving (HAD) in light grey.

Figure 2 (top) represents the percentage of different scan paths observed relative to the theoretical number of all possible permutations of AOIs for scan path length 2 to 8. The curve has an elbow at a scan path length of four, indicating that the increase of the theoretical number of possible permutations of AOIs stops translating into different scan paths in the data. In more detail, the number of different scan paths observed depending on the scan path length (Fig. 2, bottom), is still increasing but much more slowly than the exponentially increasing number of theoretical permutations of AOIs.

Scan path length of four seems to offer the longer sequence of transitions between AOIs while giving a manageable number of distinct scan paths. However, we hasten to point out that a scan path length of four should not be considered as a proposal for a *general* standard. Rather, the suitable length of the scan path should be adjusted depending on the data and the situation analysed.

### Scan path distributions

**Figure 3:**
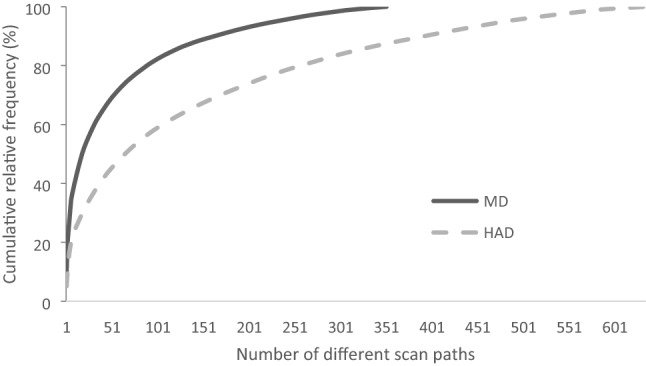
Cumulative relative frequency distribution of the different scan paths, of length four, observed under Manual Driving (MD) and Highly Automated Driving (HAD).

351 different types of scan paths of length four are present in the data for Manual Driving (MD) and 634 in Highly Automated Driving (HAD). Under HAD, scan paths were thus much more diverse. The five most frequent scan paths represent respectively about 31% and 18% of all scan paths observed in MD and HAD (Fig. 3). About 40% and 24% are accounted for by the ten most frequent scan paths, about 69% and 45% by the fifty most frequent, and about 89% and 67% by the one hundred and fifty most frequent scan paths (see Fig. 3).

This indicates that a limited number of very frequent scan paths represents most of the sequences of visual exploration, while the rest is made up by very infrequent scan paths. A cut-off threshold for a scan path to be included in the subsequent content-based classification of the most frequent scan paths was set at 0.4% relative frequency, resulting in 50 different scan paths being considered in MD, and 45 in HAD. Together these scan paths represent 69% of the complete scan path distribution in MD and 43% in HAD. This threshold was established empirically to ensure the best compromise between two competitive objectives (i) include as many scan path combinations as possible, so that the data subset would be as representative as possible of the complete data set and (ii) reduce the number of scan paths combinations analyzed as much as possible, to reduce the complexity of the dataset and allow functional interpretations.

Note that none of the most frequent scan paths analyzed were composed of four different AOIs. That is, all the scan paths included at least one *repetition*, where gaze returns to an AOI previously visited within the scan path. In MD 62.92% of the analyzed most frequent scan paths were composed of only two different AOIs and 37.08% of three different AOIs. In HAD 60.07% were composed of only two different AOIs and 39.93% composed of three different AOIs.

To complete the scan paths distributions description, and based on the complete dataset, the total number of scan paths and the number of scan paths performed only once were analyzed participant by participant. In MD, on the average, each participant performed a total of 100.44 scan paths (± 49.24) among which 42.38 (± 18.13) appeared only one time. In HAD, on the average, each participant performed a total of 116.44 scan paths (± 57.65) among which 66 (± 35.21) appeared only one time.

## Results (Part I)

### Scan paths classification

The different scan paths observed were classified according to the AOIs previously described (see Fig. 1). Because the number of distinct scan paths was fairly small, we were able to classify the scan paths via manual inspection and then to devise a taxonomy of scan path classes. This was done iteratively based on previous knowledge gained on visual exploration of the driving scene while steering on open roads see^44^ and during a car-following task. While performing a car-following task, drivers are known to devote about 40% of their visual fixations to the leading vehicle^3^ and from slightly above that percentage to gaze at the leading vehicle^19^ to up to 80% of the time spent at looking the leading vehicle^15^ depending on the driving context. As a consequence, many of the scan paths were expected to involve the leading vehicle.

Along with this expectation, and based on the three levels unified visuomotor framework described below^44^, it was hypothesized that three main classes of scan paths should emerge from the collected data. Scan paths involving visual exploration required for:

- (a) trajectory planning: look ahead fixations combined with guiding fixations, a visual strategy referred as “*gaze polling*”^45^. In the context of the car-following task, guiding fixations were expected both on and around the leading vehicle. In terms of AOIs, as defined in the current experiment, this was expected to translate into scan paths combinations involving AOIs 1, 3 and 4 in a specific order. LAFs (AOI 4—Road ahead) were expected to occur in between GFs (AOI 1—Leader or 3—Road behind Leader) The classification rule was to include all scan paths with a visit to AOI 4 in the second or third position of the sequence (i.e. X4XX and XX4X) AND a visit to AOI 1 or 3 before and after that visit of AOI 4.
- (b) guidance control: scan paths composed of guiding fixations at the leading vehicle and guiding fixations at the road around the leading vehicle were expected^46^. This would translate in scan paths combinations involving AOIs 1 (Leader) and 3 (Road behind Leader). The classification rule was to include all scan paths with a visit to LVFs (AOI 1) or GFs (AOI 3) in the second or third position of the sequence (i.e. X1XX, XX1X, X3XX and XX3X) AND another visit to AOI 1 or 3 before and after that visit.
- and (c) stabilizing control: although described as involving peripheral vision, it is also predicted to rely upon fixations at the road near the driving vehicle^47^ and could thus engage specific visual sequences. In the car-following situation, this could translate in visual sequences involving fixations at the road near the driving vehicle and guiding fixations at and around the leading vehicle, thus involving AOIs 1, 3 and 2 in a specific order. NFs (AOI 2 -proximal road) were expected to occur in between GFs (AOI 1 or 3), and alternatively, GFs (AOI 1 or 3) were expected to occur in between NFs (AOI 2). The classification rules were to include all scan paths (i) with a visit to AOI 2 in the second or third position of the sequence (i.e. X2XX and XX2X) AND a visit to AOI 1 or 3 before and after that visit to AOI 2, (ii) with a visit to AOI 1 in the second or third position of the sequence (i.e. X1XX and XX1X) AND a visit to AOI 2 before and after that visit to AOI 1, and (iii) with a visit to AOIs 1 and 3 in the second and third positions of the sequence (i.e. X13X and X31X) AND a visit to AOI 2 before and after that visit.

This framework was used to produce an initial classification grid, from there scan path classification proceeded iteratively, in a mostly data driven manner, so as to classify all the scan paths recorded.

Two additional classes were devised based on the following set of rules. A class of scan paths engaging RSFs and NFs or LVFs with of visit to AOI 6 (RSFs, off road on the right) in the second or third position of the sequence (i.e. X6XX and XX6X) AND a visit to AOI 1 or 2 before and after that visit of AOI 6. And a class of scan paths engaging SFs (AOI 0) with GFs (AOIs 1 and 3) and NFs (AOI 2), with (i) a visit to AOI 0 in the second or third position of the sequence (i.e. X0XX and XX0X) AND a visit to AOI 1, 2 or 3 before and after that visit to AOI 0, and (ii) with two visits to AOI 0 in the first and last position (i.e. 0XX0) with fixations to AOIs 1, 2 or 3 in between.

**Figure 4:**
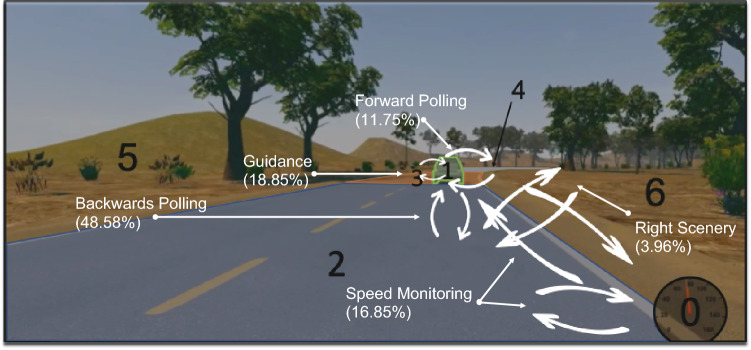
Representation of the main visual sequences identified under Manual Driving (and associated relative frequencies in percentage). Photograph JN.

Under MD, five main classes finally emerged from the different scan paths observed and hereafter referred as: (a) *forward polling* scan paths, (b) *guidance* scan paths, (c) *backwards polling* scan paths, (d) *right scenery* scan paths and (e) *speed monitoring* scan paths (see Table 1 for the observed scan paths, the classification criteria, relative frequencies, description and inferred function and Fig. 4 for an overview). While classes (a), (b) and (c) are in line with our literature-driven expectations, classes (d) and (e) were introduced in order to describe the observed data as best as possible and present a novel finding in this study. Under HAD, a sixth class unobserved under MD appeared and was referred as (f) supervision scan paths (see Table 2). Scan paths sequences that did not fit in the classes devised in MD were considered as due to automation introduction and regrouped in that class.

**Table 1:**
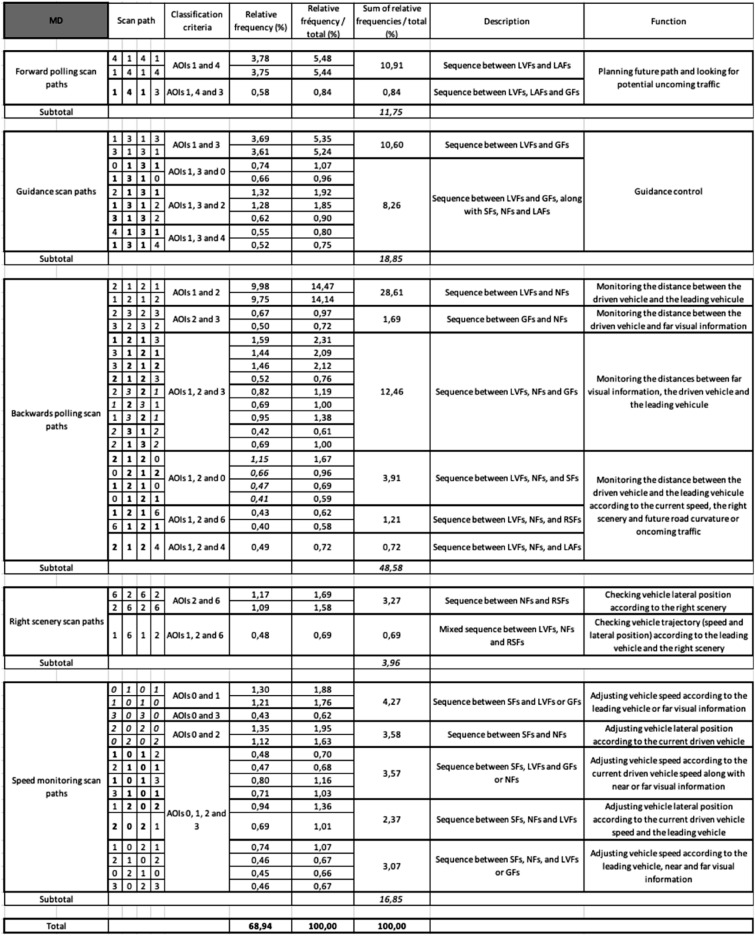
The fifty most frequent scan paths (length 4) out of the 351 scan paths observed in Manual Driving (MD). Given are each scan paths relative frequency (%) in the entire dataset, and relative frequency for the subset of the 50 scan paths analyzed (%) along with a description of the associated visual sequence and proposed functional interpretations. LVFs: Leading Vehicle Fixations; RSFs: Right Scenery Fixations; SFs: Speedometer Fixations; GFs: Guiding Fixations; LAFs: Look Ahead Fixations; NFs: Near Fixations.

**Table 2:**
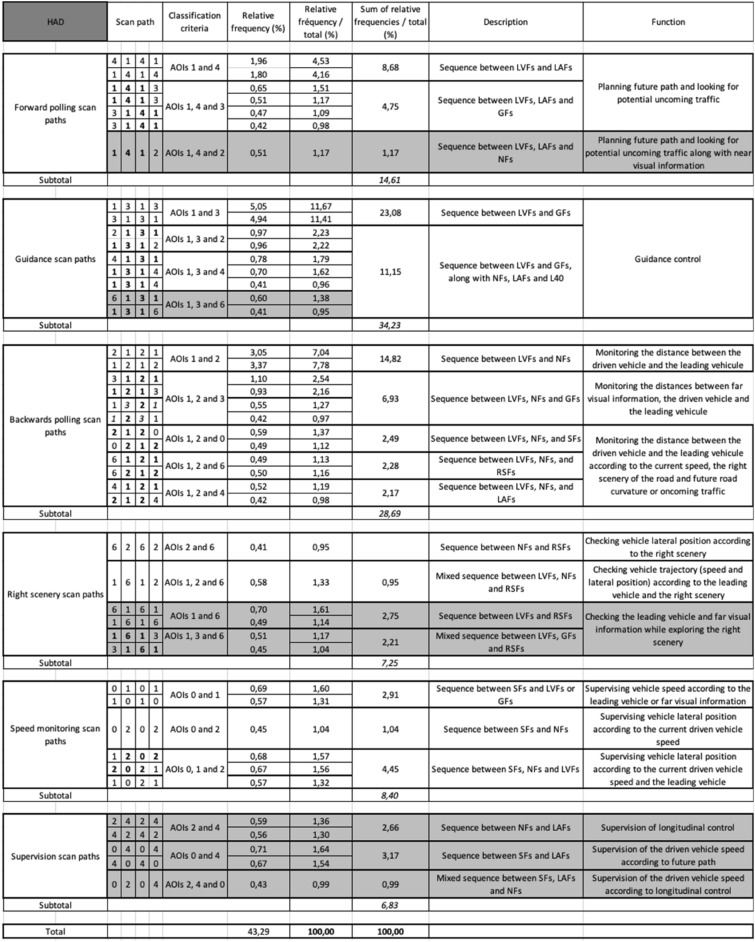
The forty-five most frequent scan paths (length 4) out of the 634 scan paths observed in Highly Automated Driving (HAD). Given are each scan path relative frequency (%) in the entire dataset, and relative frequency for the subset of the 45 scan paths analyzed (%) along with a description of the associated visual sequence and proposed functional interpretations. Scan paths in grey were not observed under Manual Driving (MD). LVFs: Leading Vehicle Fixations; RSFs: Right Scenery Fixations; SFs: Speedometer Fixations; GFs: Guiding Fixations; LAFs: Look Ahead Fixations; NFs: Near Fixations.

In order to make sure that each participant provides an equal contribution to the overall results, all the scan paths frequencies reported hereafter were computed participant per participant and then averaged.

### Manual driving

**Figure 5:**
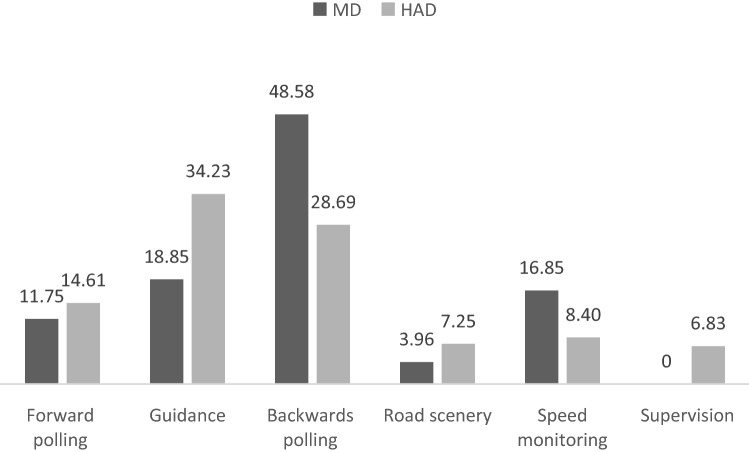
Percentage of scan paths of length 4 in the different visual sequence classes under Manual Driving (MD) and Highly Automated Driving (HAD).

Table 1 shows the fifty most frequent scan paths observed in MD. The terminology used for scan path classes will be discussed in “Interpretations of manual driving (MD) scan paths” section. The main visual sequences classes identified were not equally frequent (χ^2^ (4) = 25.01, *p* < 0.001; Fig. 5): forward polling scan paths (11.75%), guidance scan paths (18.85%), backwards polling scan paths (48.58%), right scenery scan path (3.96%).

Figure 4 represents the main visual sequences identified.

### Highly automated driving

Table 2 presents the 45 most frequent scan path sequences observed under HAD. 40 scan paths out of the 45 most frequent scan paths fall into the same five main classes observed in MD. The remaining 5 scan paths did not fall into those classes, thus a sixth class referred as supervision scan paths was added.

Tested against the null hypothesis of a uniform distribution, the observed frequencies of the six classes of visual sequences identified were significantly different (χ^2^ (5) = 19.94, *p* < 0.002; Fig. 5). Forward polling scan paths represented 14.61% of the scan paths analyzed, guidance scan paths 34,23%, backwards polling scan paths 28.69%, right scenery scan paths 7.25%, speed monitoring scan paths 8.40% and supervision scan paths 6.83%.

### Highly automated driving compared to manual driving

The distribution of scan paths frequencies across the different visual sequence classes identified was significantly different between MD and HAD (χ^2^ (5) = 20.51, *p* < 0.002; Fig. 5). A Chi-square homogeneity test, contrasting MD and HAD, for each visual sequences class, revealed a significant difference for guidance, backwards polling and supervision scan paths (χ^2^ (1) = 6.06, *p* < 0.04; χ^2^ (1) = 8.35, *p* < 0.03; and χ^2^ (1) = 7.07, *p* < 0.03 respectively), and no significant differences for speed monitoring, forward polling and right scenery scan paths (*p* > 0.05), in all cases the Benjamini–Hochberg procedure for correcting multiple comparisons was used^42^. Because all the expected values were not superior to 5 for supervision scan paths comparison, Fisher's exact test of independence was run and also revealed a significant difference (*p* < 0.02).

## Discussion (Part I)

While driving, gaze rotates around the visual scene to scan different objects and locations of interest. This results in scan paths that are fairly stereotypical in that a fairly small number of frequently recurring sequences account for a large proportion of the scan path data. These stereotypical sequences, moreover, can be classified in a fairly small number of categories that can be given a functional interpretation. Here, we were able to classify scan paths (based on dynamical AOIs) in a car-following task with lateral and longitudinal control into *forward polling*, *guidance*, *backwards polling*, *right scenery* and *speed monitoring* scan paths. Highly automated driving revealed an additional *supervising* scan path class. This represents a very substantial reduction of the gaze data in the complex dynamic task of driving. The reported scan paths analyses open new perspectives to understand not only the regions of interest on the driving scene but also the sequences of visual explorations. In the following paragraphs, the scan path categories are discussed in terms of their likely underlying perceptual-cognitive functions, according to the literature.

### Interpretations of manual driving (MD) scan paths

*Forward polling scan paths* correspond to a visual strategy initially described as an alternation between LAFs and GFs in a laboratory-based slalom steering task^45^. This visual strategy was also observed during natural driving on open roads^11^. The current results reinforce the robustness of this visual sequence between LAFs and GFs, which we find to occur in a car following task, with a leading vehicle present. In the car-following task, looking at, and around, the leading vehicle could be considered as guiding fixations^23^, and LAFs are thus fixations further along the road, beyond the leading vehicle. The current results indicate that forward polling scan paths accounted for about 12% of the scan paths analysed under MD. This visual sequence mostly engaged LVFs and LAFs (AOIs 1 and 4) and only a single scan path engaged GFs located around the leading vehicle (AOI 3).

In line with previous work, we posit that forward polling scan paths could be used by drivers to plan the future control actions^48,49^, and in particular future trajectory^18,32,44,45^. Even if no opposite traffic was present in our experiment, another function of forward polling scan paths could be to anticipate potential oncoming traffic^32^.

*Guidance scan paths* correspond to a main visual sequence between the leading vehicle and the road section around the leading vehicle. This sequence was found to engage LVFs and GFs exclusively for 10.6% of the scan paths analysed, or in combination with either SFs, NFs or LAFs for 8.26% of the scan paths analysed. These scan paths could be interpreted as visual fixations aiming at guidance control^21,46^. Alternation between LVFs and road surface GFs (AOI 1 and 3) reinforces the idea that not all the guidance relevant visual information is located at a single point in the visual scene as sometimes suggested Tangent Point Strategy^7^, but that there are rather multiple potential guiding fixation targets in the visual scene located in that far region of the road^11,17,23,43,46,50^. Also note that looking at the leading vehicle could have been used by drivers so as to track (relative) speed changes for longitudinal control cf.^24,25^ as much as a far guidance point for steering^23^.

Guidance control scan paths were also found associated with three other task relevant locations in the driving scene, namely the speedometer (AOI 0), the near section of the road (AOI 2) and the very far section of the road (AOI 4) but systematically associated with a back and forth sequence between LVFs and GFs (AOI 1 and 3). This suggests that guidance control is closely linked to both near and very far road sections and speed control. Indeed, while guidance control is often described as exclusively operational, in line with the terminology introduced by Michon^13^, we would suggest that guiding fixations may also contribute to more tactical task aspects such as the consideration of the current speed and future path planning.

*Backwards polling scan paths* correspond to a sequence between LVFs/GFs and NFs. This class was named backwards polling to refer to drivers making a glance back from the far region to the roadway between the leading and the driven vehicle. It is assumed here that this visual sequence mostly aims at monitoring the distance between the leading and the driven vehicle. The presence of a leading vehicle to follow is known to capture drivers’ visual attention by attracting drivers’ visual fixations^3,15^. However, this "near-road" polling sequence between the leading vehicle and the near road section has not been described previously.

Backwards polling scan paths could also be linked to lateral control monitoring or monitoring the road surface that becomes visible from under the leading vehicle may be another relevant function (especially in the real world with more unpredictable road surface and real consequences of hitting a pothole). Monitoring of lateral position is traditionally assumed to be undertaken using peripheral vision^3,51,52^ and presented as so in vision science models^20,44,47^. But of course, if gaze performs backwards polling fixations anyway (for LV distance or road surface monitoring), then of course information from these fixations can *also* be used for stabilizing lateral control.

As described above, both AOI 1 and AOI 3 could be classified as guiding fixations, but the decision to separate those two regions was made in order to account for the extra information that could be extracted from AOI 1. Indeed, on top of guidance control, looking at the leading vehicle also provides information on the leading vehicle speed and thus direct information to adjust the driven vehicle speed. The results indicate that most of the backwards polling visual sequence occurs between LVFs and NFs (alone = 28.61%, with SFs, RSFs or LAFs: 3.91% + 1.21% + 0.72% = 5.84%). In addition, this back and forth sequence between LVFs and NFs was found to be associated with GFs (12.46%), resulting in a total of 46.91% of all the scan paths analysed. A small amount of scan paths (1.69%) between GFs and NFs was also associated to backwards polling to monitor the distance between the driven vehicle and far visual information. For the car-following task used in this experiment, backwards polling scan paths seems to be highly relevant as a total of 48.58% of the total number of scan paths are devoted to that visual sequence.

In sum, gaze polling, guidance and monitoring scan paths extend to car-following the three levels visuomotor framework of the control of steering and gaze^44^. In this framework, look ahead fixations are assumed to support trajectory planning, a function that according to the reported data, would be supported by forward polling scan paths. In the model, guidance control is assumed to rely on far visual information. Here, LVFs and GFs represent far visual information, and the guidance scan paths observed are thus directly in line with the model predictions. Guidance scan path were also found associated to speed monitoring, near and very far road sections (AOIs 2 and 4) and the right scenery. This data tends to indicate a direct link between guidance and those areas of the driving scene. Backwards polling would represent the third level of the model referred as stabilizing control (this could be both longitudinal. via LV distance, and lateral, via future path near point). This level of control, while it *can* be performed under peripheral vision^52^, would not rely *only* on peripheral vision, but rather on a visual sequence between the leading vehicle and the near road section. This behaviour may be more prominent in the car-following task used in the reported experiment. Following a vehicle that continuously change speed engaged drivers to monitor that vehicle location on the road relatively to the driven vehicle in order to adjust the distance between the two vehicles.

Two additional classes also emerged from the scan paths analyses. *Right scenery scan paths* correspond to a sequence between RSFs and NFs, occasionally combined with LVFs. The visual control of driving is not just about the asphalt surface the vehicle will drive on. Participants engaged scan paths with looks at the right scenery. These looks are assumed to be related to driving either to check the lateral position of the vehicle or to look for potential hazards.

It is worthwhile to note that no scan paths engaging looks at the left side of the road were found. As if this region of the driving scene was not relevant for the car-following task at hand. A possible explanation would be that drivers considered the left lane of the road as offering a safety margin toward potential hazards located on the left side of the road^53^. If such an interpretation need to be tested in future experiments, the absence of scan paths including looks at the left scenery reinforce the driving relevance of RSFs rather than landscape exploration looks.

*Speed control scan paths* correspond to visual sequences engaging SFs and LVFs/GFs and/or NFs. These scan paths could be interpreted as devoted to adjusting the vehicle speed according to the leading vehicle, the road curvature to come and the lateral position on the lane. If driving speed is known to concentrate visual fixations^54^ and speedometer use variable^55^, fixations at the speedometer are usually considered as simple speed checks. The quite frequent (16.85%) speed monitoring scan paths analysed argue for a deeper implication of speedometer fixations in visuo-motor control.

Both right scenery and speed monitoring scan paths are not present in the models of vision control while driving^12,20,23^ that have aimed to primarily capture the sensory-motor nature of steering control, also referred as operational level^13,56^. In addition models proposed to perform a car-following task tend to assume that the visual scene directly provides ego vehicle speed information^24,25,57^. However, steering a vehicle also implies a number tactical decisions engaging brain regions associated with higher levels of cognitive control^14^. Scan paths including looks at the right scenery and at the speedometer may be devoted to support tactical decisions, about speed and lateral position, depending on several factors such as potential hazards on the right side of the road or current speed of the vehicle for instances.

### Interpretations of highly automated driving (HAD) scan paths

In line with previous observations^19,43,58,59^, visual explorations under HAD were not completely different from those observed in MD. The five scan paths classes identified in MD were also observed in HAD. That said, their relative frequencies were different and the scan paths inside those classes were not exactly the same. In addition, a sixth class of scan paths was added to better describe the different scan paths recorded.

*Forward polling scan paths* were very similar to those in MD, with a back and forth sequence between the leading vehicle and look ahead fixations. An insignificant frequency increase (almost 3%) of these scan paths was observed. Compared to MD, four additional exemplars of scan paths corresponding to the forward polling scan paths category were observed (7 scan paths combinations in HAD instead of 3 in MD) including a new exemplar with near fixations.

*Guidance scan paths* were significantly more numerous in HAD compared to MD (HAD: 34.23%, MD: 18.85%). Guidance is described as preview information from far visual information in order to specify the driven vehicle future path^12,20,44,47^. This indicates that under HAD participants keep on performing guidance control and even increase the frequency of that control as compared to MD (relative frequency increase of about 15%). Thus, under HAD drivers not only keep on performing visual sequences hypothesized to contribute to specifying the future path, but even reinforce that visual sequence. This indicates that drivers in our study were still engaged in the car-following task, despite the full delegation of steering and longitudinal control to automation. The result is in line with real driving eye movements collection with drivers looking at the forward roadway about 75% of the time with HAD or without^60^.

*Backwards polling scan paths* frequency significantly decreased during HAD, compared to MD (HAD: 28.69%, MD: 48.58%), and the number of different scan paths in this class also dropped from 20 to 12. These results extend previously collected data indicating that automated steering and HAD decreased the amount of visual attention devoted to near looks at the road^19,43,58^. Indeed, it is not only the number of near road glances that decrease, but specifically the backwards polling visual sequences. This could be interpreted as a disengagement from monitoring the distance between the leading and the driven vehicle (or near road surface or lateral position), because in HAD the "out of the loop" driver is anyway not even able to quickly adjust the driven vehicle current speed (or road position).

*Right scenery scan paths* were not significantly more frequent under HAD as compared to MD (HAD: 7.25%, MD: 3.96%) but twice the number of scan paths were collected in HAD (HAD: 6, MD: 3).

*Speed control scan paths* were less frequent under HAD although not significantly different from MD (HAD: 8.4%, MD: 16.85%), and the number of scan paths also dropped from 15 to 6. Speed supervision visual sequence as defined for MD, tend to be less important under HAD.

*Supervision scan paths* correspond to visual sequences engaging LAFs along with NFs, SFs or both. These scan paths unobserved under MD could support automation supervision especially for longitudinal control with a sequence between near and/or speedometer fixations and look ahead fixations. To our knowledge, such specific visual supervision behaviours have not been reported so far.

Only scan paths not observed in MD have been included in this new class. However, automation supervision could also take place within the classes already existing in MD. Indeed, the same sequences could have been used to supervise automation rather than perform the driving task. This interpretation even seems obvious as no driving control per se was required in the HAD condition. Interestingly drivers did not simply engage visual sequences in HAD as they do in MD, rather they favour guidance scan paths over backwards polling and speed monitoring scan paths. In other words, drivers favoured far visual information over near visual information. Drivers were thus engaging a more anticipative strategy, as if they were looking for potential automation failure they could correct manually. However, it must be noted that backwards polling scan paths were not completely dropped, but rather performed less frequently. In brief, HAD transform the nature of the car-following task from a the mostly operational task into a more tactical one, where automation supervision takes more importance.

## Results (Part II)

In the visual sequences previously analysed, very often participants were found to visit an AOI already visited in the same visual sequence, with just a visit to another AOI in between (41 scan paths out of 50 in MD and 42 out of 45 in HAD; see Tables 1, 2). Complementary analyses were undertaken in order to further investigate these back and forth scan paths of length three. Based on the previous findings, the purpose of these additional analyses was to (i) extend the analyses to the complete set of data, (ii) use previous findings to generate a set of pre-defined scan paths, (iii) simplify the scan paths classes by using a given region of interest as the middle of the sequence and (iv) reduce scan path length to the shortest meaningful sequence.

Based on the previous analysis, the following set of scan paths was pre-defined:

The *forward polling class* was found to be largely based on a back and forth sequence between AOIs 1 and 4 (i.e. scan path 141). Along with this scan path, the three other permutations between guiding fixations on the leading vehicle (LVFs) or on the road (GFs) and LAFs, as an AOI visited in between (i.e. 143, 343, 341), were included.

The *guidance class* was largely based on a back and forth sequence between AOIs 1 and 3 (i.e. scan path 131). The other permutation between those two AOIs (i.e. 313) were added so as to constitute the guidance class.

The *backwards polling class* was mostly based on a back and forth sequence between AOIs 1 and 2 (i.e. scan path 121). The three other permutations, also centered on NFs (AOI 2) and engaging guiding fixations on the leading vehicle (LVFs) or on the road (GFs) were included (i.e. 123, 323, 321).

The *right scenery class* was based on back and forth sequences between looks at the road and looks at the right scenery, i.e. AOIs 1/3 and 6 and AOIs 2 and 6.Thus, the four permutations between RSFs and guiding fixations on the leading vehicle (LVFs) or on the road (GFs) were considered (i.e. 363, 161, 361, 163) along with the back and forth visual sequence between NFs and RSFs (i.e. 262). In addition, all the combinations of NFs and GFs or LVFs, with the right scenery visited in between, were included in that class (i.e. 263, 261, 362, 162).

The *speed monitoring class* was mostly based on back and forth sequences between AOIs 1 and 0 and AOIs 2 and 0. Thus, the four permutations between SFs and guiding fixations on the leading vehicle (LVFs) or on the road (GFs) were considered (i.e. 303, 101, 301, 103) along with the back and forth visual sequence between NFs and SFs (i.e. 202).

The *supervision class* was based on a back and forth sequences between AOIs 2 and 4 and AOIs 0 and 4 (i.e. scan paths 242 and 040). Those two permutations plus two additional permutations between NFs and LAFs, with speedometer visited in between, were considered (i.e. 402, 204). As such supervision scan paths are not following the rule of a single region of interest being in the middle of the sequence, with LAFs and SFs being used as such. This choice was made to avoid the division of supervision scan paths in several subclasses.

All in all, 28 scan paths sequences of length three (referred to as triplets, below), derived from the six classes from the initial analysis, were investigated further, in terms of their incidence in the entire data set (as opposed to the reduced data set of most frequent length-four scan paths used in the initial analysis and classification).

In MD, on the average, each participant performed a total of 101.44 scan paths (± 49.24) among which 28.25 (± 11.72) appeared only one time. In HAD, on the average, each participant performed a total of 117.44 scan paths (± 57.65) among which 43.44 (± 15.01) appeared only one time.

**Figure 6:**
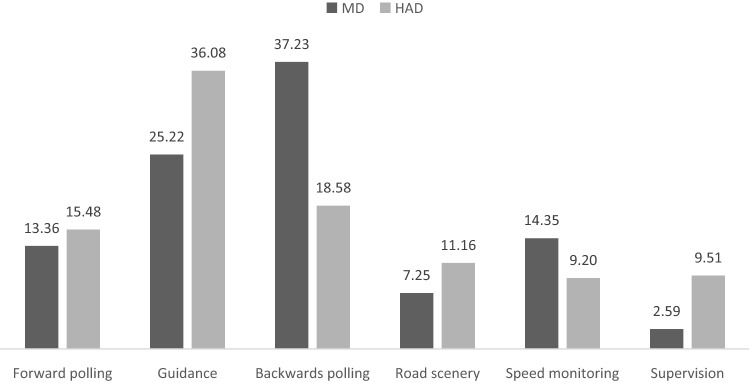
Percentage of scan paths of length 3 in the different visual sequence classes under Manual Driving (MD) and Highly Automated Driving (HAD).

The absolute frequency of the 28 triplets represent about 45% of the entire scan paths sequences dataset in MD and about 38% in HAD (Table 3). The observed frequencies of the six classes of predefined visual sequences were significantly different for MD (χ^2^ (5) = 24.13, *p* < 0.001) and HAD (χ^2^ (5) = 12.49, *p* < 0.03; Fig. 6).

**Table 3:**
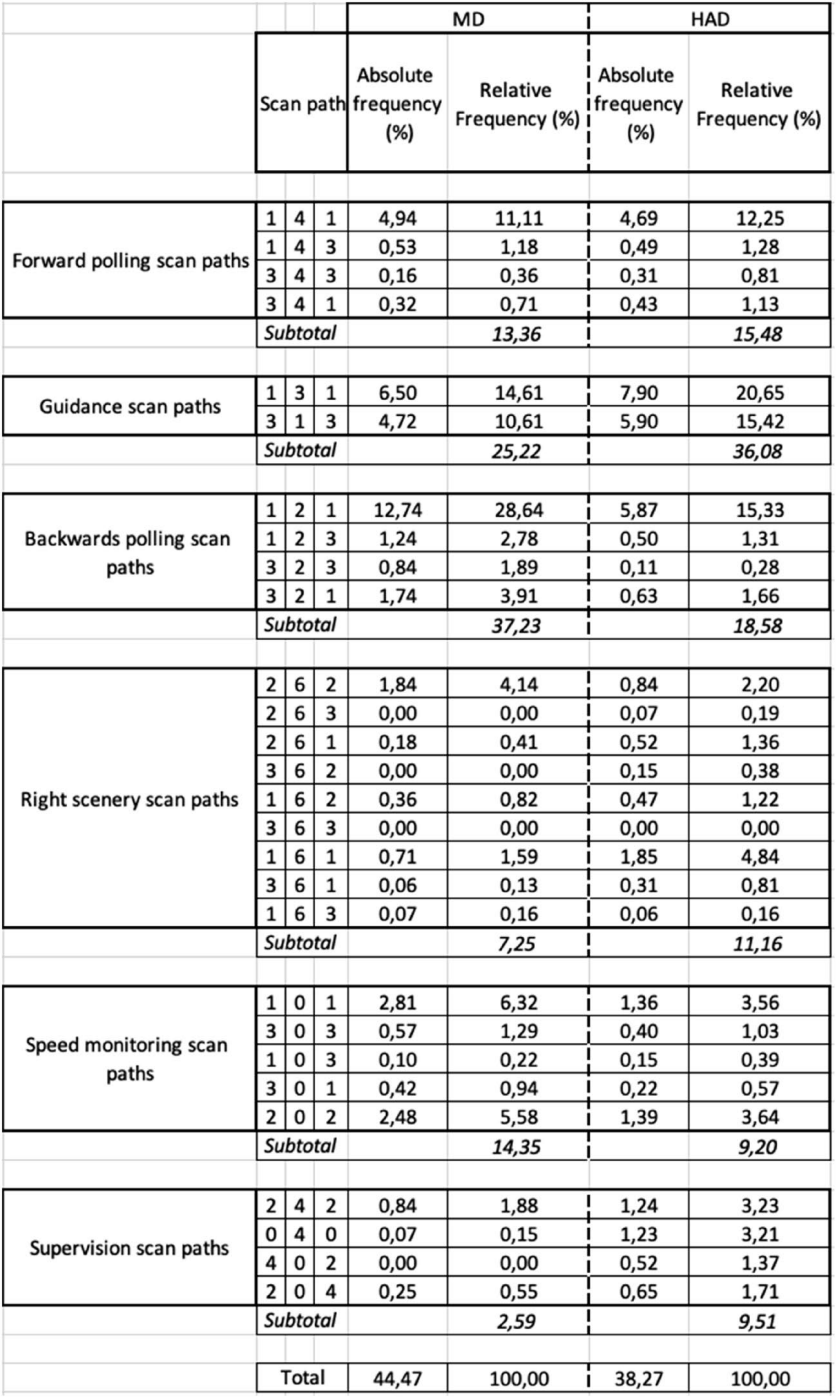
Absolute and relative frequencies of the 28 triplet scan path sequences derived from the six classes in Manual Driving (MD) and Highly Automated Driving (HAD). Given are each scan path absolute frequency (%) in the entire dataset, and relative frequency for the subset of the predefined 28 scan paths sequences (%).

The distribution of scan path frequencies across the different visual sequence classes identified was significantly different between MD and HAD (χ^2^ (5) = 14.23, *p* < 0.02; Fig. 6). Chi-square contrasting MD and HAD, for each visual sequences class, revealed a significant difference for backwards polling (χ^2^ (1) = 8.64, *p* < 0.02), but no significant differences for supervision, forward polling, guidance, right scenery, and speed monitoring (*p* > 0.05), in all cases the Benjamini–Hochberg procedure for correcting multiple comparisons was used^42^.

The distribution of scan path frequencies observed for the predefined scan paths length of three did not differ significantly from those collected for the previous analysis of scan path length 4 for both MD (χ^2^ (5) = 6.28, *p* = 0.28) and HAD (χ^2^ (5) = 3.54, *p* = 0.62).

## Discussion (Part II)

### Additional analysis findings

In the initial analysis and classification, only the most frequent, rather than all the observed combinations of visual sequences, were considered. While prior literature on driver visual strategies were used in defining scan paths classes, careful search for patterns in the results pointed out the existence of additional classes to report, and account for the recorded data.

In the initial analyses, a partial exploration of a data subset of manageable size was necessary in the absence of accurate pre-defined classes of scan paths to be expected. Based on these analyses of the subset of data containing the most frequent (length four) scan paths initially analysed, we identified recurring triples, i.e. length-three scan paths with just a visit to another AOI in between (these comprised more than 87% of all the scan paths initially analysed). Such an observation suggested a special functional relevance for those visual sequences. We then went on to look for the incidence of the identified triplets in the complete set of data. The main observation was that fairly similar results were collected between the distributions of scan paths frequencies across the different classes for the initial and second analysis, and so for both MD and HAD. Despite the reduction of the sequence length from 4 to 3 AOIs, the use of pre-define scan paths and the consideration of the complete dataset, no significant differences were found between the initial and the second analysis. This suggests that those shorter sequences may be sufficient to describe much of the main visual strategies used by drivers, at least to perform a car-following task.

These results overall fit with, and refine, the three levels unified visuomotor framework of the control of steering and gaze^44^. This integrative perspective has three levels of steering control—trajectory planning, guidance control and stabilizing control—with each level being supported not by specific visual cues. Here, it is proposed that those three levels of visual control of steering should not only be described in terms of visual cues from a single fixation, but rather on sequential patterns of extended visual sequences.

Regarding trajectory planning, drivers are making back and forth visual sequences, from guiding fixations at and around the leading vehicle, to glances to the furthest visible section of the road. Those latter look ahead fixations were initially defined as “*not relevant to the immediate sub-task, but relevant for a future sub-task*”^49^ and usually associated with trajectory planning^18,32^. In line with previous observations, it is proposed here that forward polling visual sequences are meant to plan ahead the future trajectory and/or anticipate traffic in line^45^. Forward polling was found to represent between 12 and 15% of the visual sequences depending on the driving condition and analysis. The relative frequency of forward polling is thus very consistent between MD and HAD.

The guidance control level aims at seeking for information located on the path defined at the previously described trajectory planning level. Guidance control level was found to rely on guidance scan paths combining looks at the far region of the road and on the leading vehicle, consistent with the idea that drivers look at waypoints on the future path^45,61,62^ or the tangent point^7,21^. Guidance scan paths include looks at the road, but also at the leading vehicle favoring the idea that not all the steering relevant information is located at a specific point of the driving scene. In manual driving, guidance scan paths accounted for about 19% and 26% respectively in the first and second analysis. Guidance scan paths were even more frequent in highly automated driving and represent about 35% of the different scan paths analyzed.

Finally, stabilizing control is a control loop aiming at adjusting the vehicle trajectory^47,63^. In terms of visual cues, stabilizing control is presumed to rely on peripheral vision^52,64^. If peripheral vision might contribute to stabilizing control, the results reported here showed that while following a vehicle under manual driving backwards polling scan paths, combining looks at the near road and at far regions, were the most frequent of all scan paths analyzed. Suggesting that stabilizing control also engages a large amount of foveal visual sequences. Interestingly, a large reduction of backwards polling scan paths was observed under HAD, as if drivers partly disengaged from scan paths required to ensure stabilizing control when automation handle steering. Ironically, HAD puts drivers in a situation where the vehicle is undertaking steering control, but at the same time expect the driver to manage that same steering control if automation fails to do so^34^. Drivers are thus expected to keep monitoring the road in order to deal with potential return to manual control. Even if longer eyes-off-road durations were measured^60^, visual explorations under HAD were not found to be tremendously changed by the introduction of driving automation in a series of previous experiences^19,43,58^. Our current experiment flagged a significant reduction of backwards polling scan paths under HAD, precisely the visual sequences associated to stabilizing control.

Another clear change triggered by HAD was spotted in the percentage of different scan paths observed relative to the theoretical number of all possible permutations of AOIs (see Fig. 2). Whatever the scan path length (from 2 to 8), more numerous scan paths were observed in HAD compared to MD. This finding indicates that drivers’ visual exploration sequences were more diverse under HAD. Although non-significant, some of the extra scan paths recorded under HAD were found to be dedicated to automation supervision (i.e. supervision scan path). It can be hypothesized that other extra scan path might not be dedicated to vehicle control but rather to non-driving related tasks such as the exploration of the landscape for instance.

### Limitations of the present work

The reported experiment also comes with a number of limitations. Obvious limitations are that the task was only carried out in a simulator, so correspondence to real world behavior remains open, and that the task only lasted for a relatively short amount of time, so effects of learning or boredom remain uncertain. Future experiments are also required to better describe the automation supervision processes, in particular to describe how automation is impacting visual sequences over longer periods of time^65^.

Investigations of visual sequences engaged in other driving scenarios would also be of interest. In line with automation, investigations of scan paths related to a particular event such as in case of automation take-over request while driving under highly automated driving would allow a better understanding of the visual processes engaged by drivers in such situations^38^. The scan path methodology could also benefit to lower levels of automation such as warnings (i.e. lane departure warnings or forward collision warnings) to analyse quantitatively the spatio-temporal visual strategies used by drivers^36^.

Apart from automation, here the focus was set on car-following, a recurring driving situation used to analyze the following visual and/or locomotor performances under a variety of conditions^66–68^. This driving situation produced a number of visual sequences that may differ from the visual sequences while driving on an open road, in cities, with or without traffic, with drivers being distracted or not, experts or novices, etc. More investigation is required to examine the influence of the driving environment, the driving context and individual differences.

Another important limitation of the reported data is the unexplored visual sequences because they were infrequent in the first analysis or outside the scan path classes in the second analysis. Those leftovers scan paths represent a notable number of drivers visual scanning sequences (about 31% and 57% in MD and HAD respectively for the first analysis and about 56% and 62% for the second analysis). Even if not all fixations, and sequences of fixations, are necessarily meaningful in everyday life activities^69^, further investigations of all possible visual sequences, over short periods of time for instance, would be sound.

The large amount of collected data represented a challenge for data analysis. A number of methodological choices have been made along the analysis process. Alternative methodological choices may have resulted in different outputs. The scan path classification proposed is key to offer a reading grid of the scan paths combinations recorded. It offered (i) the possibility to consider an important number of scan paths combinations and (ii) proposed a framework that organize the collected scan paths in relation to driving. The classification was devised based on a combination of previous knowledge about visual behaviours engaged while driving and data-driven observation. Thus, in future investigations, knowledge or methodologies might lead to reconsider the proposed categories. Alternative methodologies to devise scan paths clusters could be foreseen, based on automatic clustering methods for instance^70^. To facilitate that process, all the scan paths combinations and relatives’ frequencies were made available (Tables 1, 2, 3). Another important methodological choice was to focus on the visual sequences without considering the duration of the fixation per se. Our rationale was that the order of visual fixations would provide insights on the cognitive processes engaged by the driver that time analysis (e.g. time spent gazing at AOIs) would not. In other words, investigating the order of visual fixations would provide information of how drivers organize their visual explorations, that is to say in which order the AOIs of the visual scene are considered. As a matter of fact, time information is not discarded, as the order of visual fixations is at the heart of the analyses, it is rather processed on an ordinal scale. A perspective of improvement would be to consider the duration of fixations within the scan paths. Such additional information could help to weight the relative importance of the visual sequences. Finally, the methodology used does not investigate how the different scan paths identified are arranged over time. Further experiments targeting the inter-relations between the visual sequences identified would be welcome. Such investigations should seek for recurring distribution patterns of the different scan paths classes over time and depending on the driving context.

## Conclusion

Regarding manual driving, the analyses allowed us to identify and classify recurring scan path sequences between AOIs. We interpret the scan paths in light of the control of steering and gaze framework that synthetized more than 50 years of research on driving^44^. However, that work was based on investigation of individual fixations/gaze targets, and their relative frequencies. We feel it is important to extend experimental work and models of visual strategies to sequential structure in gaze behaviour (e.g. based on scan path analysis methods such as those used in this study).

Based on the collected data, we propose trajectory planning is supported by forward polling scan paths, guidance control by guidance scan paths and stabilizing control by backwards polling scan paths. In addition, two other main visual sequences have been identified: right scenery and speed monitoring scan paths. Those can be interpreted as tactical visual sequences potentially able to interact with all three levels of the control of steering, speed and gaze.

Regarding highly automated driving, the analyses revealed that drivers remained engaged in similar visual sequences as those required to drive manually. However, the frequency of those different sequences was impacted with an increase of guidance scan paths and a decrease of backwards polling scan paths. In addition, automation specific scan paths were recorded, and placed in a class named supervision scan paths, in reference to automation supervision.
